# Objects tell us what action we can expect: dissociating brain areas for retrieval and exploitation of action knowledge during action observation in fMRI

**DOI:** 10.3389/fpsyg.2014.00636

**Published:** 2014-06-24

**Authors:** Ricarda I. Schubotz, Moritz F. Wurm, Marco K. Wittmann, D. Yves von Cramon

**Affiliations:** ^1^Institute for Psychology, University of MünsterMünster, Germany; ^2^Max Planck Institute for Neurological ResearchCologne, Germany; ^3^Department of Neurology, University Hospital CologneKöln, Germany; ^4^Center for Mind/Brain Sciences (CIMeC), University of TrentoMattarello, Italy; ^5^Department of Experimental Psychology, University of OxfordOxford, UK

**Keywords:** fMRI, object perception, action observation, apraxia, affordance, pantomime

## Abstract

Objects are reminiscent of actions often performed with them: knife and apple remind us on peeling the apple or cutting it. Mnemonic representations of object-related actions (action codes) evoked by the sight of an object may constrain and hence facilitate recognition of unrolling actions. The present fMRI study investigated if and how action codes influence brain activation during action observation. The average number of action codes (NAC) of 51 sets of objects was rated by a group of *n* = 24 participants. In an fMRI study, different volunteers were asked to recognize actions performed with the same objects presented in short videos. To disentangle areas reflecting the storage of action codes from those exploiting them, we showed object-compatible and object-incompatible (pantomime) actions. Areas storing action codes were considered to positively co-vary with NAC in both object-compatible and object-incompatible action; due to its role in tool-related tasks, we here hypothesized left anterior inferior parietal cortex (aIPL). In contrast, areas exploiting action codes were expected to show this correlation only in object-compatible but not incompatible action, as only object-compatible actions match one of the active action codes. For this interaction, we hypothesized ventrolateral premotor cortex (PMv) to join aIPL due to its role in biasing competition in IPL. We found left anterior intraparietal sulcus (IPS) and left posterior middle temporal gyrus (pMTG) to co-vary with NAC. In addition to these areas, action codes increased activity in object-compatible action in bilateral PMv, right IPS, and lateral occipital cortex (LO). Findings suggest that during action observation, the brain derives possible actions from perceived objects, and uses this information to shape action recognition. In particular, the number of expectable actions quantifies the activity level at PMv, IPL, and pMTG, but only PMv reflects their biased competition while observed action unfolds.

## Introduction

Observed action entails a highly complex stimulus that prompts a multitude of attentional and memory processes. The observer has to be flexible with regard to potential actions that may unroll, but yet quickly discard those which do not pertain to the actual situation. How is this achieved?

When considering object-related action, the observer has access to at least two sources of information that usually help him to quickly recognize the most probable action goal: manipulation movements and objects. These two basic sources of information, rather than being complementary, are intimately interrelated: familiar objects such as mobile phones or knifes are strongly reminiscent of manipulations that we perform with them everyday. Hence, the observer's brain may use these automatically evoked memories of distinct object-related actions (action codes, hereafter) to bias or constrain expectation on upcoming manipulations and hence facilitate recognition of the action, i.e., implemented object function, and thereby the probable actor's goal. For instance, when seeing someone handling a knife and an apple, the object set “knife, apple” evokes two action codes: “cutting apple with knife” and “peeling apple with knife.” While tracking the unfolding manipulation, we at a point in time notice that the peeling-action code matches the observed manipulation, and recognize the actor is peeling the apple with the knife (object function), probably to prepare it for eating (goal).

The present fMRI study focused on automatically evoked object-related action codes to find out if, and if so how, they influence the neural basis of action observation. In order to recognize an observed action, it would make no sense to match the observed action to *all possible* action memories we have. Rather, one could suggest that objects automatically evoke mnemonic codes of the handful of actions we most frequently perform with them (i.e., action codes) (Helbig et al., 2006; Myung et al., 2006; Campanella and Shallice, 2011), information that could greatly constrain the number of expectable actions. Note that we very quickly recognize objects (Bar, 2003), including their pragmatic properties (Liu et al., 2009; Proverbio et al., 2011), while observed manipulation only unfolds and disambiguates in time.

Although the notion of action codes is reminiscent of what is called object *affordance*, they differ in an important respect. According to the classical concept of affordance (Gibson, 1977; see McGrenere and Ho, 2000 for modifications and alternatives), an object affords actions in that seeing the object can automatically prime, and hence facilitate, object-compatible actions in a particular observer (depending also on the observer's body); the object does so by virtue of its physical properties: e.g., size and shape of the object afford appropriate grasping, and its location appropriate pointing (Tucker and Ellis, 1998, 2001, 2004; Craighero et al., 1999; Pavese and Buxbaum, 2002; Phillips and Ward, 2002; Derbyshire et al., 2006; Symes et al., 2007; Cho and Proctor, 2010; Pellicano et al., 2010; Iani et al., 2011; McBride et al., 2012). In contrast, we here were interested in object-evoked representations of actions that do not derive from the object's size, shape or orientation, but from associative memories of how and what for we use these objects in everyday life. Note that our manipulation did not dissociate this “how” and “what for,” which can be doubly dissociated in patient groups (Buxbaum and Saffran, 2002).

**Figure 1:**
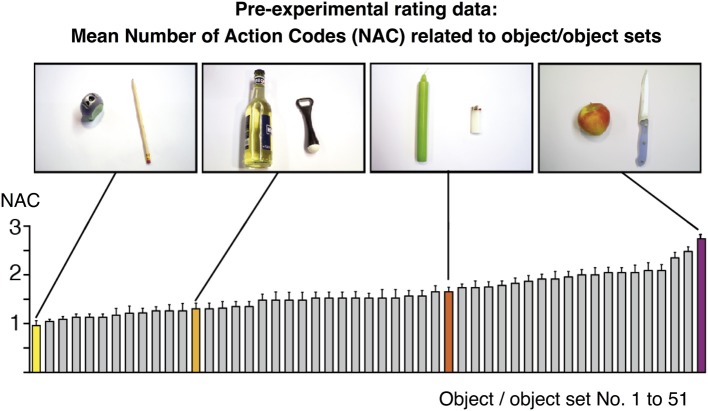
**Rating values for the number of action codes (NAC) related to 51 objects/object sets as assessed pre-experimentally in an independent group of *n* = 24 volunteers**. Values were subsequently used to model the BOLD fMRI amplitude during object-compatible and object-incompatible action. Some values are highlighted and correspond to the objects shown in the photos, based on which participants delivered their ratings. For instance, the object set “pencil and sharpener” were mostly reported to be related to “sharpening a pencil” (resulting in NAC=1), whereas “candle and lighter” participants often associated “lighting a candle,” and sometimes also “melting a candle's foot” (in order to firmly fixate it in a candle stand) (NAC=1.65).

To show that action codes are effective during action observation, we should find that it makes a difference how many action codes are currently evoked, and whether the observed action matches one of them or not. Accordingly we should find (H1) increased activity in areas that code for currently active action codes; and (H2) increased activity in areas that exploit them for action recognition. In order to disentangle these effects, we presented object-compatible (normal) action and object-incompatible (pantomime) action. As an example for an incompatible action, the actor performed the movements for “cracking an egg” while holding and moving an orange and an orange squeezer. Object-compatible and object-incompatible actions were performed on objects whose NACs, i.e., number of possible action codes related to them, were assessed in a pre-fMRI rating study (see Methods and Figure 1).

The NAC effect (H1) should be only driven by the perceived object(s) but be independent of the actually observed action. Thus, we considered areas that positively co-vary with the NAC during object-compatible and object-incompatible action to classify as areas storing action codes. In contrast, currently evoked action codes can only be exploited for action recognition (H2) when observing the former, but not the latter. That means, only if the observed actor executes one of the currently evoked action codes, i.e., in object-compatible actions, can the observer benefit from their automatic pre-activation.

Thus, these action codes put an effective constraint on the to-be-expected possible actions, and identification of the matching action will be enhanced. In terms of neural computation, this results in a continuous, top-down reinforcement of the matching action code, in competition to all currently active action codes, during ongoing action observation (see neuroanatomical hypotheses below). This reinforcement may be achieved by enhancement of the matching action code, or by inhibition of the currently competing but non-matching action codes, or both. Since the present approach could not distinguish between these options, we will refer to this mechanism shortly as “reinforcement” hereafter.

It is particularly essential that, in order to interpret an area's activation as exerting a reinforcement of one particular action code among all currently evoked and hence competing action codes, rather than simply signaling for a successful matching, this activation has to depend on competition strength: to make the particular matching action code to come out on top of three possible actions (NAC 3) is more demanding than on top of two actions or only one (NAC 2 or 1, respectively). Accordingly, regarding (H2), we were not interested in the main effect of object compatibility, but rather in the *interaction* between the NAC and object compatibility: We considered areas that positively co-vary with the NAC during object-compatible *significantly more than* during object-incompatible action to classify as areas exploiting the currently active action codes. These areas should show a significant parametric effect of NAC in object-compatible actions, no significant parametric effect of NAC in object-incompatible actions, and a significant interaction between the NAC and object compatibility.

Regarding the neural correlates of action code storage (H1), we hypothesized that activity in the left anterior inferior parietal lobule (aIPL) increases with the NAC, no matter whether the movie shows an object-compatible or an action-incompatible manipulation. During tool-related tasks, left aIPL is often seen in co-activation with left ventral premotor cortex (PMv) and the posterior middle temporal gyrus (pMTG) (Johnson-Frey, 2004; Culham and Valyear, 2006; Martin, 2007; Creem-Regehr, 2009), i.e., exactly the same network that is reported for action observation (Grèzes and Decety, 2001; Van Overwalle and Baetens, 2009; Caspers et al., 2010), but also for action execution, action imagery, action planning, and action imitation. This network has been referred to as MNS (mirror neuron system) or AON (action observation network), but due to the spectrum of action-related roles of this triad, the label “Action Network” might be more generic. Regarding our hypothesis on areas housing action codes (H1) we focused on aIPL because of converging findings from various studies reporting left aIPL to be engaged in the representation of pragmatic properties of objects, particularly manipulation knowledge (e.g., Chao and Martin, 2000; Kellenbach et al., 2003; Johnson-Frey, 2004; Rumiati et al., 2004; Boronat et al., 2005; Ishibashi et al., 2011). In her thorough review, Creem-Regehr (2009) proposed to conceive of the inferior IPL/IPS as a region for motor cognition, including the generation of internal representations for action and knowledge about actions. Patient studies indicate that the ability to retrieve the correct manipulation for a given tool can be selectively impaired, while in the same patient, the ability to correctly name the tool or point to the tool when named by the experimenter are preserved (Ochipa et al., 1989). This defect in tool utilization has been coined limb apraxia (Rothi and Heilman, 1997). In spite of considerable variance between findings, evidence converges that patients with impaired object use and pantomiming to visually presented objects mostly suffer from lesions that include the left IPL (Rumiati et al., 2004). This region is considered crucial for gestural praxis, tool knowledge, body part knowledge, and manipulation knowledge, together coined as the ability to generate internal models of object-interaction (Buxbaum et al., 2005).

The frontal component of the Action Network, the left PMv, was expected to respond quite differently than aIPL. In relation to our second question, whether there would be areas reflecting the selection among competing action codes, we hypothesized (H2) the left PMv to be enhanced by the number of action codes, but in contrast to aIPL only for object-compatible, not object-incompatible action videos. That is, we should see left PMv only for the interaction between NAC and object compatibility of the observed action.

This hypothesis was motivated by the notion that premotor regions serve the top-down selection among alternative manipulation options provided in parietal areas (Fagg and Arbib, 1998; Rushworth et al., 2003). The lateral premotor cortex is made of a variety of functionally highly specialized sub-areas which in turn are connected in multiple parallel, largely segregated loops to a mosaic of sub-areas making of the parietal cortex (Luppino and Rizzolatti, 2000). Among these premotor-parietal loops, the ventral premotor—inferior parietal loop was reported to code for grasping and manipulation (Rizzolatti et al., 1987), but also for the sight of graspable objects (via so-called canonical neurons in PMv, Murata et al., 1997; Rizzolatti and Fadiga, 1998). As for fronto-parietal loops in general, the functional role of PMv with regard to IPL is providing inhibitory and reinforcing input to focus and elevate currently relevant codes in IPL to modulate adaptive perception, attention and behavior.

Addressing the interplay between lateral premotor and parietal areas in object-directed action, Fagg and Arbib (1998) put forward that anterior parietal cortex provides ventral premotor cortex with a multiple description of how the object can be grasped and used. In PMv, then, all corresponding motor acts are first activated, and then the currently required one is selected (or reinforced, to keep with the more process-dynamic notion adopted above). For instance, neurophysiological studies in macaques indicate that potential plans for movements to multiple targets are simultaneously represented in parietal and frontal areas (Andersen and Cui, 2009) and, as information accumulates, eliminated in a competition for overt execution (Cisek and Kalaska, 2005) (for an application of the notion of selection as frontoparietal reinforcement signal in humans, see e.g., Ramsey et al., 2013). Fagg and Arbib (1998) proposed that in action execution, this selection needs prefrontal input (via pre-supplementary motor area) that signals the current goals of the individual. However recent imaging findings indicate that action selection that emerges from the race between competitive decision-units is reflected in premotor, not prefrontal, areas (Rowe et al., 2010), suggesting that action selection in premotor sites does not necessarily need prefrontal bias.

In the present experimental approach, competition between the action codes evoked by the perceived object was to be resolved by the actually observed manipulation. We expected that in case of a successful match (which was only possible for object-compatible actions), the PMv would reinforce the matching action codes in aIPL, just as it does during action execution. Load on this reinforcement would be a function of action codes only in object-compatible action, as outlined above, manifesting in an interaction of the NAC and object compatibility of the observed action. Of course, if PMv does exert an action code dependent reinforcing signal on aIPL during action observation, this effect should be reflected in both of these areas.

Finally, in object-incompatible action, reinforcement load should be generally higher than in object-compatible action, as action recognition is unrestricted by the currently evoked action codes: there are objects that evoke action codes, but none of them matches the observed action. That does also mean, reinforcement load should *not* depend on the number of action codes in case of object-incompatible actions. Since objects employed in object-incompatible actions evoke action codes that are not effective to constrain the matching process, the number of possible actions is the number of all possible actions that humans do perform with objects. Accordingly, our third hypothesis (H3) was that object-incompatible actions lead to an overall higher response than object-compatible actions in left PMv and IPL (replicating Schubotz and von Cramon, 2009), but show no positive co-variance with the number of currently activated action codes.

## Materials and methods

### Participants

Seventeen right-handed, healthy volunteers (13 women, 20–31 years, mean age 25.6 years) participated in the study. After being informed about potential risks and screened by a physician of the institution, participants gave informed consent before participating. The local ethics committee of the University of Cologne approved the experimental standards. Data were handled anonymously.

### Stimuli and tasks

**Figure 2:**
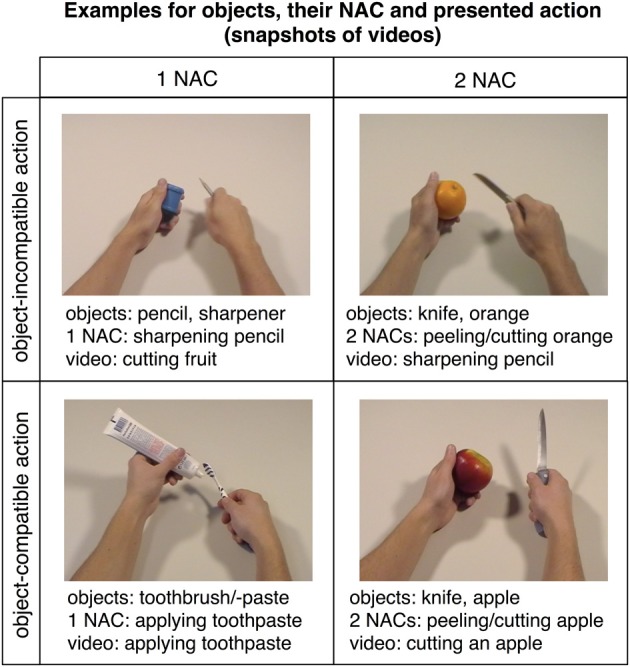
**Snapshots of videos showing actions with objects mostly related to either one (left panel) or two (right panel) actions**. Actions could be exploited to constrain action recognition only in object-compatible actions (lower panel; examples show “applying toothpaste” on the left and “cutting an apple” on the right), but not in object-incompatible actions (upper panel; examples show “cutting a fruit” on the left and “sharpening a pencil” on the right). The corresponding videos can be found in the supplementary material.

**Figure 3:**
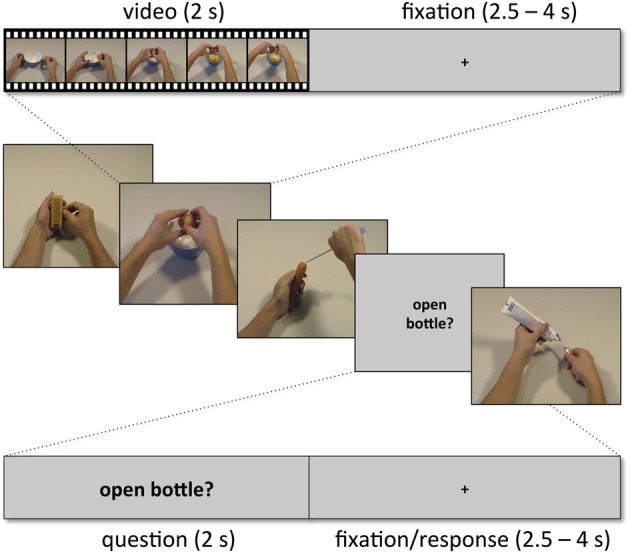
**Example sequence of five trials with within-trial time course for one video trial (top) and for one question trial (bottom)**.

Subjects were presented with two kinds of trials, videos showing actions (snapshots in Figure 2; for examples of videos, see supplementary material) and short verbal action descriptions (without video) referring to these actions (e.g., “cutting bread,” “peeling an apple,” “cleaning a cell phone”). Each trial lasted 6 s and started with a movie (2 s) followed by a fixation phase (Figure 3). The length of the fixation phase (2.5–4 s) depended on the variable jitter times (0, 500, 1000, or 1500 ms) that were inserted before the movie to enhance the temporal resolution of the BOLD response. Actions were either performed on appropriate objects (object-compatible actions, e.g., peeling an apple with a knife) or on inappropriate objects (object-incompatible actions, e.g., making the same movements with a pencil and a sharpener).

Subjects were instructed to attend to the presented movies. They were informed that some of the movies were followed by a trial providing a verbal action description that either matched or did not match the content of the preceding movie. Subjects then performed a verification task, i.e., they were asked to indicate by button press whether the verbal description was consistent with the action movie previously presented or not. It was emphasized that it did not play any role whether actions, to which the action description referred, were object-compatible or not. Thus, when subjects saw the action “peeling an apple” performed with an apple and a knife, or with a pencil and a sharpener, and the subsequent trial delivered the verbal action description “peeling an apple,” the correct answer was “yes.” In the case of an action description trial, participants immediately delivered their responses on a two-button response box using their index finger for affirmative responses (description pertains to the movie in the preceding trial) and their middle finger for rejections (description did not pertain to the movie in the preceding trial). Half of the action descriptions were to be affirmed and half to be rejected.

Action movies varied with regard to the number of action codes (Figure 1; see also “Pre-experimental assessment on objects' average number of action codes”). Importantly, in case that two or three objects were involved in an action, they always made up object sets that were indicative of possible actions (which were, of course, not actually performed in the case of object-incompatible actions); e.g., participants were presented the object-incompatible action “cracking an egg” performed in an as-if manner on the objects “orange” and “orange squeezer,” i.e., a pair of objects that could be used to prepare orange juice (Figure 2). Thus, videos showing object-incompatible actions never involved meaningless object sets such as e.g., an orange and a sharpener.

Twenty percent of the movies (i.e., 21 of 105 object-compatible actions and 21 of 105 object-incompatible actions) were followed by an action description that had the length of a regular trial (2 s description, including response phase, plus 4 s fixation phase), resulting in 42 additional trials. Finally, 20 empty trials (resting state) of 6 s duration were presented intermixed with the experimental trials. Thus, 272 trials were presented altogether.

For each subject, each action was presented four times during the course of the experimental session, two times object-compatible and two times object-incompatible, with different objects each time. Importantly, we balanced the order of appearance of object-compatible and incompatible actions in the time course of the appearance. Hence, all combinations (1: compatible, compatible, incompatible, incompatible; 2: compatible, incompatible, compatible, incompatible; 3: incompatible, compatible, incompatible, compatible; 4: incompatible, incompatible, compatible, compatible) occurred equally often in the experiment.

### Pre-experimental assessment on average number of action codes

In order to determine the NACs of the objects later used in the action movies, we assessed the spontaneous assignment of actions to these objects in a group of *n* = 24 volunteers. To avoid mnemonic confounds, this group was not identical to the group tested in the fMRI session, i.e., none of the participants of the pre-experimental assessment was included in the fMRI study. Participants were given photographs of 51 objects (e.g., cell phone) or object sets (e.g., apple and knife). There were 27% single objects, 63% two-object sets and 10% 3-object sets. Participants were asked to write down all potential actions that the presented objects were typically reminiscent of in their eyes. For instance, participants rated an apple and a knife to be most suggestive of “cutting an apple into halves,” “peeling an apple,” and “coring an intact apple” (3 actions), whereas an orange and an orange squeezer were rated suggestive of “squeezing an orange” (1 action). To assess NACs rather than object affordances, participants were explicitly asked to provide object-specific goal-directed actions, not object grasping or transport.

We did not impose a temporal restriction onto this rating process and participants had time to thoroughly ponder on potential actions. The collection of actions typically took less than 1 min per object or object set; moreover, no participant came up with invalid or odd actions. On the basis of this rating, the average NAC score was calculated for each object or set of objects (Figure 1). NAC scores, ranging from 0.95 to 2.73, were subsequently used in the parametric analysis of fMRI data (see below). Importantly, there was no systematic relation between NAC score and number of objects in a set. Thus, single objects yielded a mean NAC of 1.63 ± 0.33, two-object-sets 1.66 ± 0.4, and three-object sets 1.18 ± 0.1. To statistically rule out the potential confound that NAC co-vary with the number of objects displayed in an action, we calculated a correlation of NAC with the number of objects. There was no correlation [*r*_(49)_ = −0.223, *p* = 0.12].

### MRI data acquisition

Twenty-two axial slices (192 mm field of view; 64 × 64 pixel matrix; 4 mm thickness; 1 mm spacing; in-plane resolution of 3 × 3 mm) parallel to bi-commissural line (AC–PC) covering the whole brain were acquired using a single-shot gradient EPI sequence (2 s repetition time; 30 ms echo time; 90° flip angle; 116 kHz acquisition bandwidth) sensitive to BOLD contrast. Prior to the functional imaging, 26 anatomical T1-weighted MDEFT images (Ugurbil et al., 1993; Norris, 2000) with the same spatial orientation as the functional data were acquired. In a separate session, high-resolution whole-brain images (160 slices of 1 mm thickness) were acquired from each participant to improve the localization of activation foci using a T1-weighted 3-D-segmented MDEFT sequence covering the whole brain.

### fMRI data analysis

After offline motion-correction using the Siemens motion protocol PACE (Siemens, Erlangen, Germany), fMRI data were processed using the software package LIPSIA (Lohmann et al., 2001). To correct for the temporal offset between the slices acquired in one image, a cubic-spline interpolation was employed. Low-frequency signal changes and baseline drifts were removed using a temporal high-pass filter with a cutoff frequency of 1/90 Hz. Spatial smoothing was performed with a Gaussian filter of 5.65 mm FWHM (*SD* = 0.8 voxel). To align the functional data slices with a 3-D stereotactic coordinate reference system, a rigid linear registration with six degrees of freedom (three rotational, three translational) was performed.

The rotational and translational parameters were acquired on the basis of the MDEFT slices to achieve an optimal match between these slices and the individual 3-D reference dataset. The MDEFT volume dataset with 160 slices and 1-mm slice thickness was standardized to the Talairach stereotactic space (Talairach and Tournoux, 1988). The rotational and translational parameters were subsequently transformed by linear scaling to the same standard size. The resulting parameters were then used to transform the functional slices employing a trilinear interpolation, so that the resulting functional slices were aligned with the stereotactic coordinate system. Resulting data had a spatial resolution of 3 × 3 × 3 mm (27 mm3).

The statistical evaluation was based on a least-squares estimation using the general linear model for serially auto-correlated observations (Friston et al., 1995; Worsley and Friston, 1995). The design matrix was generated with a delta function, convolved with the hemodynamic response function (gamma function) (Glover, 1999). The design matrix comprised the following events: object-compatible action videos, object-incompatible action videos, object-compatible action videos with an amplitude modeled by the corresponding objects' NAC, object-incompatible action videos with an amplitude modeled by the corresponding objects' NAC, question trials, and empty trials (null events).

Brain activations were analyzed time-locked to onset of the videos. The model equation, including the observation data, the design matrix, and the error term, was convolved with a Gaussian kernel of dispersion of 4 s FWHM to account for the temporal autocorrelation (Worsley and Friston, 1995). In the following, contrast images, that is, beta value estimates of the raw-score differences between specified conditions were generated for each participant. As all individual functional datasets were aligned to the same stereotactic reference space, the single-subject contrast images were entered into a second-level random effects analysis for each of the contrasts.

One-sample *t*-tests were employed for the group analyses across the contrast images of all participants that indicated whether observed differences between conditions were significantly distinct from zero. The *t*-values were subsequently transformed into *z*-scores. To correct for false-positive results, an initial *z*-threshold was set to 2.33 (*p* < 0.01, one-tailed). In a second step, the results were corrected for multiple comparisons at the cluster level, using cluster size and cluster value thresholds that were obtained by Monte-Carlo simulations at a significance level of *p* = 0.05, i.e., the reported activations were significantly activated at *p* ≤ 0.05, corrected for multiple comparison at cluster level.

## Results

### Behavioral results

Performance was assessed by error rates and reaction times. We calculated paired-*t*-tests (one-tailed, in expectation of lower performance in object-incompatible actions) for each of these measures between question trials addressing object-incompatible and object-compatible actions. While error rates became not significant (*t*_16_ = −0.66, *p* = 0.259), reaction times showed a small effect (*t*_16_ = 1.75, *p* = 0.049). Thus, object-compatible and object-incompatible actions were responded to equally correct (mean ± SE: object-compatible action 5.8 ± 1.2% errors and object-incompatible 4.8 ± 1.4% errors), but recognition of object-incompatible actions took 40 ms longer (object-compatible action 1192 ± 61 ms and object-incompatible 1232 ± 69 ms).

Moreover, we calculated bivariate correlations between NACs and reaction times or error rates, respectively. As a result, there was neither an effect on error rates [*r*_(16)_ =− 0.012, *p* = 0.96], nor on reaction times [*r*_(16)_ = −0.026, *p* = 0.92]. Together, behavioral statistics suggested that recognition times were slightly but significantly reduced by object information, but both object-incompatible and object-compatible actions could be successfully identified.

As a caveat, we employed a retrospective judgment in order to control for the participants' performance in action recognition. This task was implemented by extra question trials that followed an action observation trial in order to avoid response-related confounds: motor execution, and, even worse, trial-specific interactions between executed button press, implied and observed manipulations. That is, reaction times and error rates refer to a response delivered one trial after action observation. So, our paradigm was optimized for fMRI rather than for specific behavioral effects.

### fMRI results

Our two main hypotheses H1 and H2 addressed the parametric effect of number of action codes. In left aIPL, this effect was expected to be independent of the object-compatibility of the observed action (Hypothesis H1), but depend on object-compatibility in PMv (Hypothesis H2). H1 was tested by calculating a conjunction of the thresholded parametric contrast in object-compatible actions and the thresholded parametric contrast in object-incompatible actions. H2 was tested by an interaction contrast, i.e., by contrasting the parametric effect of number of action codes in object-compatible actions with the parametric effect of number of action codes in object-incompatible actions. While H2 addressed compatible > incompatible actions, we also report the reverse contrast for exploratory reasons. We follow the view that in order to show that differences between the two parametric effects (number or action codes in object-compatible actions, number of actions in object-incompatible actions) are statistically significant, it is not enough to show each of them, but to calculate a contrast between both, i.e., an interaction (Nieuwenhuis et al., 2011). In order to make their respective contributions to these two effects descriptively transparent, however, we also report the parametric effect in object-compatible actions and the parametric effect in object-incompatible actions separately. Finally, we report findings on the main effect of object compatibility of observed action (Hypothesis H3).

#### Parametric effect of number of action codes (NAC) common to object-compatible and object-incompatible actions (hypothesis H1)

**Figure 4:**
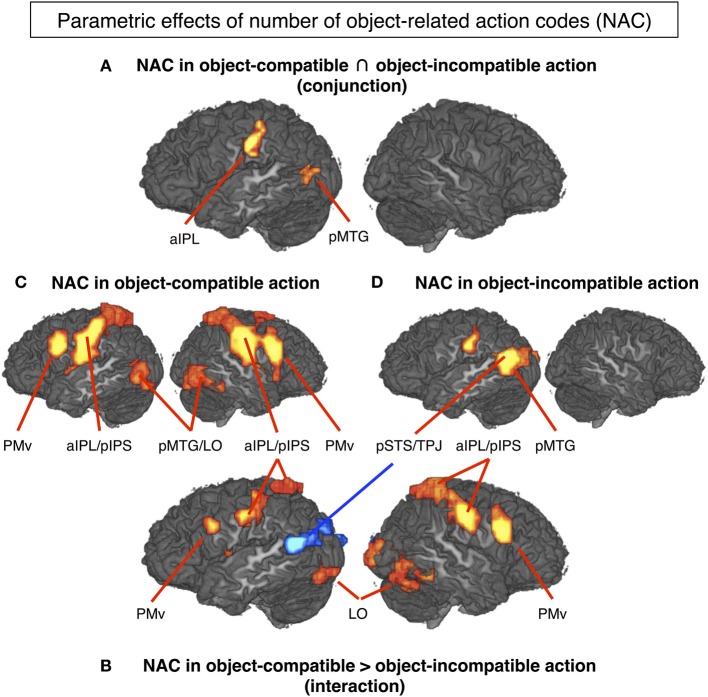
**The mere sight of objects triggered the representation of a number of possible object-related actions that quantified the activation in several cortical areas (*P* < 0.05, corrected for multiple comparisons)**. **(A)** Left aIPL and pMTG can be considered to code for the number of object-related action codes (NAC), as their activity increased with the NAC regardless of whether the observed action was object-compatible (and hence matched one of the evoked NACs) or object-incompatible (cf. Hypothesis H1). **(B)** In contrast, PMv, right IPS, left pIPS, bilateral LO and mid-insula increased with NAC only in object-compatible action, presumably reflecting a top-down competition bias between the observed and the remaining object-related but unobserved actions (cf. Hypothesis H2). Interestingly, left pSTS/TPJ increased with NAC in object-incompatible actions blue spot in **(B)** suggesting that in case of a non-match, lower constraints on expectable actions (i.e., higher NACs) increased efforts to read out the actor's hand postures and movements. For descriptive purposes, **(C)** and **(D)** show the NAC effect separately for videos on object-compatible and object-incompatible (pantomime-with-incompatible-objects) actions. Table 1 lists Talairach coordinates for *z*-maps shown in **(A)** and **(B)**, Table 2 for **(C)** and **(D)**.

The conjunction between the thresholded parametric effect of action codes in *object-compatible* actions and the thresholded parametric effect of action codes in *object-incompatible* actions revealed activation in the left aIPS and in the left posterior middle temporal gyrus (pMTG). Both areas showed increasing activation with the number of possible actions associated with the objects shown in the movies (Figure 4A, Table 1).

**Table 1:** **Anatomical area (for abbreviations, see main text), Talairach coordinates (*x, y, z*) and maximal *Z*-score (max) of activated clusters (*p* = 0.05, corrected for multiple comparisons) for parametric effects of the number of automatically evoked object-related action codes (NAC) that were common to both for object-compatible and object- incompatible actions (conjunction; Hypothesis H1) or interacted with object compatibility of observed action (Hypothesis H2) (cf. Figures 4A,B)**.

**Area**	***x***	***y***	***z***	**Max**
**H1: NAC IN BOTH OBJECT COMPATIBLE ∩ INCOMPATIBLE ACTION**				
aIPS	−62	−24	36	3.08
pMTG	−47	−66	9	2.83
**H2: INTERACTION NAC *x* OBJECT COMPATIBLE > INCOMPATIBLE ACTIONS**				
PMv	−56	9	27	3.08
	52	9	24	4.43
aIPS	−50	−27	36	4.18
	40	−27	42	5.22
pIPS	−35	−54	63	3.66
	31	−45	66	4.61
Mid-Insula	−38	−6	21	3.24
	34	−3	34	3.42
LO / pMTG	37	−69	−3	4.07
Cuneus	22	−90	6	5.10
pSTS / TPJ	−59	−57	15	−3.65
Cuneus	−8	−81	0	−4.81

#### Interaction effect of NAC and object-compatibility (hypothesis H2)

***Parametric effect of NAC in object-incompatible actions***. For object-incompatible action, activity increased with number of action codes in the left aIPS, the left pMTG, encroaching into adjacent pSTS and TPJ, and in the left Cuneus (Figure 4D, Table 2).

**Table 2:** **Anatomical area (for abbreviations, see main text), Talairach coordinates (*x, y, z*) and maximal Z-score (*max*) of activated clusters (*p* = 0.05, corrected for multiple comparisons) for parametric effects of the number of automatically evoked object-related action codes (NAC), separately analyzed for object-compatible actions and for object-incompatible actions (cf. Figures 4C,D)**.

**Area**	***x***	***y***	***z***	**Max**
**NAC IN OBJECT-COMPATIBLE ACTIONS**				
aIPS/SMG	−47	−24	33	5.38
	49	−21	33	5.67
pMTG, LO	−47	−63	3	3.51
	43	−63	0	4.79
Fusiform gyrus / LO	−47	−75	−6	3.06
Fusiform gyrus	46	−45	−12	3.18
pIPS	−32	−51	60	5.74
	28	−48	60	5.39
	37	−30	39	5.91
PMv	−56	6	27	4.23
	52	9	27	5.29
PMd	22	−12	51	3.64
Mid-Insula	−38	3	12	4.55
	31	−6	18	4.77
**NAC IN OBJECT-INCOMPATIBLE ACTIONS**				
aIPS	−62	−24	33	3.15
pMTG/pSTS/TPJ	−53	−69	21	4.13
Cuneus	−14	−78	−3	4.59

***Parametric effect of NAC in object-compatible actions***. For object-compatible action, activity increased with number of action codes in the Action Network (PM, aIPL, pMTG) as well as the fusiform gyrus/lateral occipital cortex and mid-insula. All activation spots were found in both hemispheres (Figure 4C, Table 2).

***Interaction effect of NAC in object-compatible > incompatible actions***. For object-compatible action, additional activation was found to increase with the NAC in right ventral and dorsal premotor cortex (PMv, PMd) as well as in bilateral mid-insula. Moreover, activation in IPS was recorded bilaterally and extended from anterior into its horizontal segments. Finally, activation in the pMTG extended inferiorly and posteriorly into the lateral occipital cortex (LO) and emerged particularly in the right hemisphere (Figure 4B, Table 1).

***Interaction effect of NAC in object-incompatible > object-compatible actions***. Some areas responded to increasing number of evoked action codes exclusively during object-incompatible actions. These were located in the left pSTS, extending posteriorly and dorsally into the temporo-parietal junction (TPJ), and in left cuneus (Figure 4B, Table 1).

#### Main effects of observation of object-compatible and object-incompatible action (hypothesis H3)

**Figure 5:**
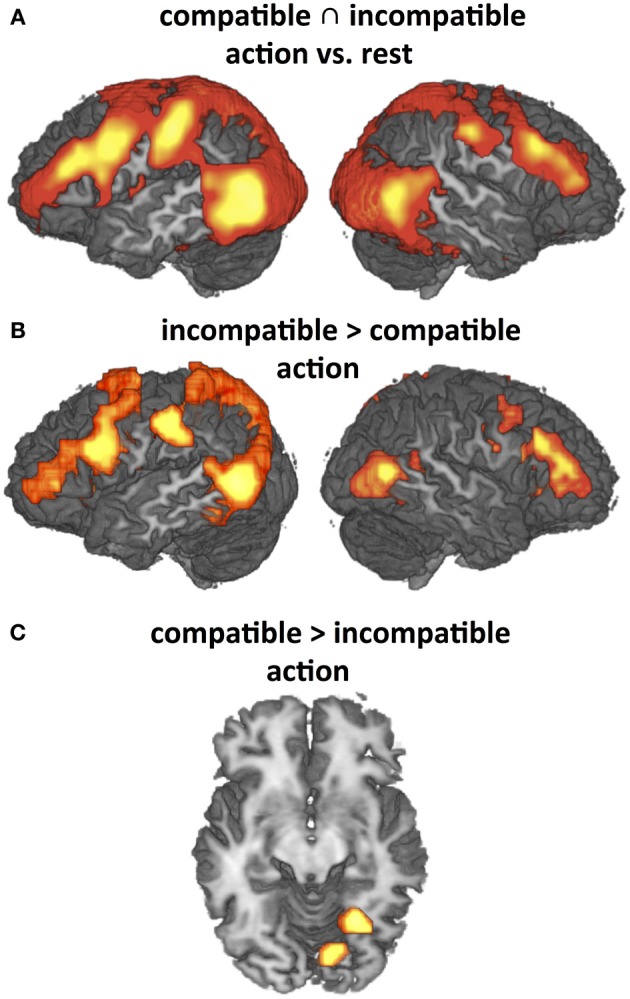
**Significant activation differences addressed by Hypothesis (H3) (*P* < 0.05, corrected for multiple comparisons) that replicate previous findings (Schubotz and von Cramon, 2009)**. **(A)** Main effects of observation of object-compatible and object-incompatible action as compared to rest (conjunction). **(B)** and **(C)**: Differential effects as revealed by direct contrasts between observation of object-compatible and object-incompatible action. Both object-compatible and object-incompatible actions induce strong activation in the Action Network, including premotor-parietal and temporo-occipital areas. This activation pattern is even intensified by object-incompatible action **(B)** where participants have to rely on manipulation information to recognize the action. Correspondingly, activity is significantly enhanced in fusiform gyrus when object information is valid **(C)**.

The present study employed object-compatible and object-incompatible action to investigate the effects of action codes and their impact on action observation. However, it is important to consider that all effects so far reported supervened on the typical network found for action observation (cf. Introduction), including the lateral premotor-parietal loops as well as temporo-occipital areas related to attention to motion, movements and objects (Figure 5A). Recorded activation patterns were almost identical for object-compatible and object-incompatible actions when compared to rest, but direct contrasts revealed significant differences in the modulations of this network as well, particularly enhanced activity in left PMv and IPL for object-incompatible actions (Figure 5B). For object-compatible actions, we found enhanced activity solely in fusiform areas (Figure 5C). These findings fully replicate those of a previous study (Schubotz and von Cramon, 2009; Figure 3).

In contrast to the findings in Schubotz and von Cramon (2009), object-incompatible actions in the current study additionally activated mesial Brodmann Area (BA) 8, the ventral tegmental area and the bilateral dorsal anterior insula (not shown in Figure 5). These activations probably reflect dopaminergic enhancement during decision uncertainty (Volz et al., 2005). We consider this difference to be due to the use of object sets that always implied valid action options for object-compatible as well as for object-incompatible actions, in contrast to our previous study. Thus, uncertainty was somewhat higher for object-incompatible, as objects did not indicate whether the to-be-expected action would be an object-incompatible or an object-compatible action, and action codes could not become effective to constrain the process of identifying the observed action.

## Discussion

Objects are reminiscent of actions that we typically perform with them. These object-related actions (action codes) may influence action observation by providing a constraint on the number of expectable actions, and hence facilitate action recognition. We used fMRI in an action observation paradigm to test whether left aIPL codes for action codes, i.e., whether its activation level varies as a function of the currently evoked number of action codes (main effect action codes; Hypothesis H1). Moreover, we employed object-compatible and object-incompatible action videos to test whether left PMv reflects the exploitation of evoked action codes. Here we reasoned that an area that exploits action codes in action observation should positively co-vary with the NAC in case of object-compatible, but not object-incompatible action, since action codes can act as a constraint only in the former (interaction effect action codes x object compatibility; Hypothesis H2).

In expectation to replicate findings from a previous study (Schubotz and von Cramon, 2009), we hypothesized that object-compatible and object-incompatible action differ in highly similar way from the resting level, but when directly contrasted with one another show enhanced activity for object-incompatible actions in the entire Action Network, including left PMv and IPL (Hypothesis H3).

### Responses to automatically evoked codes of object-related actions

Object-compatible and object-incompatible actions differed with respect to the usability of object information, but objects implied possible actions in both. To tap this object-based action-pre-activation, we computed the parametric effect of the number of action codes separately for object-compatible actions and object-incompatible actions, and subsequently built the conjunction of both. As a result, activity was recorded in only two areas, the left aIPL and the left pMTG (Figure 4A). Finding aIPL confirmed our hypothesis, which was based on the role of inferior parietal lobe in the appraisal of pragmatic implications provided by objects. Left pMTG was not hypothesized and will be discussed as a *post-hoc* finding.

The left IPL activation was restricted to the anterior bank of the intraparietal sulcus (aIPS) and did not encroach into supramarginal gyrus (SMG). This is an important observation, since these two areas have distinct functions, as implicated by research in their putative homologues in the macaque, AIP and PF, respectively (Committeri et al., 2007; McGeoch et al., 2007). The latter mediates between PMv and pSTS in a network coined “mirror neuron system” or MNS for both action observation and action execution (as lucidly outlined in Keysers and Perrett, 2004), whereas the former provides PMv with a pragmatic description of objects (Fagg and Arbib, 1998). The core difference here is that neurons in AIP already respond to objects even when not manipulated, whereas PF neurons are particularly tuned to the sight of the experimenter grasping and manipulating objects (Gallese et al., 2002).

This difference seems particularly relevant in the context of the present findings, as the conjunction contrast aimed to tap only the parametric effects of object-evoked action knowledge, *independent of the object-compatibility of the observed manipulation*. It makes perfect sense that the parametric action codes contrast did not identify SMG (as putative human PF-homolog), because activation that was caused by observation was accounted for by the main effect action vs. rest (Figure 5), i.e., it was canceled out in the parametric action codes contrast. Notably, exploitation of action codes was reflected by extension of activation into SMG that we found only for object-compatible actions, as will be discussed later. Thus, our findings perfectly corroborate the assumption of a functional dissociation or relative weighting of AIP/aIPS reflecting object-related action information and PF/SMG reflecting the observation of object manipulation.

Human and macaque data converge with regard to the manipulation-related role of anterior intraparietal cortex. The role of macaque AIP in providing pragmatic object descriptions has been related to “hand manipulation neurons” (Gardner et al., 2007a) in this region and to the encoding of context-specific hand grasping movements to perceived objects (Gallese et al., 1994; Murata et al., 1996; Baumann et al., 2009). Human left aIPL is selectively activated during the explicit retrieval of specific ways of grasping tools (Chao and Martin, 2000) and manipulating objects (Kellenbach et al., 2003). Using an interaction design implementing two cue types (naming and pantomiming) and two response triggers (objects and actions), Rumiati et al. (2004) showed that the left aIPL is particularly active for the transforming objects into skilled object manipulation. A recent fMRI study showed that activity in human aIPS reflects the relationship between object features and grasp type, as in macaques (Begliomini et al., 2007). Also paralleling macaque data, aIPS is particularly enhanced when object information is to be transferred between the visual and the tactile modality (Grefkes et al., 2002). Our results crucially extend these findings, showing that activity in aIPS increases with the mere implication of more possible actions, i.e., the more visual properties of the objects are mentally transferred to different, merely imagined tactile properties.

The present study did not distinguish between semantic/conceptual (“what”) and procedural/motor (“how to”) representations triggered by the sight of objects, and its perfectly possible that both are automatically evoked. However, there is some evidence that aIPL is more related to the “how to” knowledge related to objects. For instance, Boronat et al. (2005) asked participants to determine whether two given objects are manipulated similarly (e.g., a piano and a laptop keyboard) or serve the same function (e.g., a box of matches and a lighter). Only the left IPL was more engaged during judgments on manipulation than during judgments on object function (cf. Kellenbach et al., 2003 for parallel findings).

Patient studies support this interpretation, showing that damage to the left IPL can result in an inability to recognize and produce precise hand postures associated with familiar objects while functional knowledge of objects seems spared (Buxbaum and Saffran, 2002; Buxbaum et al., 2003). Binkofski and Buxbaum (2013) proposed that two action systems have to be distinguished in the dorsal stream: a bilateral dorso-dorsal “grasp” stream linking superior parietal to dorsal premotor sites for reaching and grasping objects based on their size, shape or orientation; and a ventro-dorsal “use” system linking inferior parietal to ventral premotor sites for skilled functional object use.

In the present study, objects varied with regard to the number of implied actions, and thereby ways to use the objects, but of course, also in the way to grasp them. Although our parametric approach—object-evoked action options—tapped a very subtle source of variance in our stimulus material (videos), this approach did not allow distinguishing between automatically evoked representations of object-related ways of manipulating, and object-related ways of grasping (i.e., affordances). However, participants were required to recognize the observed actions, and hence could not solely rely on the observed kind of grasping; rather, they had to exactly analyze the way of subsequent usage to determine the observed action with confidence. Moreover, finding PMv and aIPL to increase with the number of active action codes points to the ventro-dorsal “use” system rather than to the dorso-dorsal “grasp” stream.

Left pMTG showed up in the action vs. rest contrast, as expected, as left pMTG is mostly seen in action observation, and also for tool perception (cf. Introduction). However, just as left aIPL, pMTG was also found to positively co-vary with the number of object-implied actions (Figure 4A). Fusiform gyrus, pMTG, and aIPL are considered sensitive to the three types of information required for identification of tools: their visual form, the typical motion with which they move when we use them, and the way they are manipulated, respectively (Beauchamp et al., 2002; Mahon et al., 2007). Following this view, we suggest pMTG and aIPL both co-varied in activation with the number of active action codes, because action codes differed not only with regard to the way we use objects, but also in the way the object moves while used. For instance, when participants saw the actor handling a knife and an apple, the automatically evoked action codes included two sorts of knife manipulation, but also the corresponding two sorts of knife motion. Of course, the visual form of the knife was invariant, fitting to the fact that action codes showed no effect in fusiform gyrus. Note that other authors have put forward that pMTG rather than being a motion-coding area, represents conceptual object knowledge (Johnson-Frey, 2004; Fairhall and Caramazza, 2013). There might be also subtle regional differences in functions, as the posterior temporal region contains a variety of functionally specialized areas.

In fact, pMTG refers to an only vague macroanatomical definition of a cortical region that lies in direct vicinity of functional related areas. The peak coordinates of the left MTG in our study were at Talairach *x* = −47, *y* = −67, *z* = 9, which is nearly identical to peak coordinates of the extrastriate body area (EBA; *x* = ±47.2, *y* = −66.7, *z* = 4.7) when averaging across 13 recent fMRI studies (Downing et al., 2006a,b; Taylor et al., 2007; Myers and Sowden, 2008). Moreover, human motion selective area hMT (Greenlee, 2000; Peuskens et al., 2005) overlaps with EBA (e.g., Downing et al., 2007; Taylor et al., 2007). Although the parametric increase of activity in EBA or hMT in the current experimental design cannot be due to demands on body part and motion perception, it could reflect the range of movements and body postures associated with a given object. On the one hand, EBA's contribution in the processing of body posture (Downing et al., 2006b) could be required here as referring to typical hand postures and configurations indicative of the manipulations applicable to an object. On the other hand, hMT is engaged in the processing of complex motion patterns (Peuskens et al., 2005), but also in motion as merely implied or announced by hand postures or objects (Kourtzi and Kanwisher, 2000). Interestingly, both hMT and EBA, together with pSTS sensitive to the perception of biological motion, were found to adapt to the repetition of observed actions even when novel exemplars of object manipulation were shown, suggesting a role of these areas in the representation of the type of manipulation rather than its particular instantiation (Kable and Chatterjee, 2006). Our findings fit to this notion, as the parametric effect of action codes in pMTG and aIPL was independent of the actual observation of one of these actions (revealed by the conjunction of both).

### Exploiting object-related knowledge to recognize actions

Only object-compatible, not object-incompatible actions matched one of the action codes supposedly evoked by the sight of the involved objects. Thus, the NAC quantified the constraint imposed onto recognizing object-compatible, not object-incompatible actions: the actually observed action was one out of about one, two or three expectable actions. As hypothesized, PMv activity positively co-varied with the NAC in object-compatible but not incompatible actions. We found this activation in both hemispheres, together with corresponding activation foci in anterior IPL, bilateral lateral occipital cortex extending into pMTG in the right hemisphere, and bilateral mid-insula (Figure 4B). Activity in pIPS was bilateral, but pronounced in the right hemisphere spanning from a ventral postcentral region and anterior SMG up to the horizontal segment of the IPS. The fact that left aIPL, the area that we had found for the parametric effect of the NAC, surfaced in this interaction contrast as well indicate that it was dominant, though not specific, for object-compatible actions.

As outlined in the Introduction, we take this network to reflect the fronto-parietal reinforcement of object-implied action options while tracking the unfolding action. Here, the observed action matched one of actions the observer was expecting due to the observed object or set of objects. In this case, and only then, PMv reinforced the matching action manipulation in IPL, and the matching tool motion in pMTG. Further extrastriate visual activation located in the right cuneus may point to modulations going even further downstream.

Importantly, this interaction contrast tapped only into areas whose activation increased with the competition load between object-evoked action options. This effect was observed not only at the frontal component of the Action Network, but also at the corresponding parietal and posterior temporal sites. Thus, reinforcement increases activation also at the targets in the posterior brain. It is well-known that frontal and parietal/temporal areas interact for selective purposes in attention, with the latter providing “bottom-up” externally driven, perceptual input on which the frontal areas exert a “top-down” selective modulation for goal-directed cognition and behavior (Frith, 2001; Bar, 2003; Pessoa et al., 2003). For sure, the relevant parietal and temporal areas themselves provide highly integrated information of the stimulus. That is, they build rather a “mid” level between frontal and lower visual areas, exerting “top-down” biasing signals on the latter as well (Kastner and Ungerleider, 2001).

Premotor-parietal-temporal activation patterns during action observation have been suggested to reflect a re-activation of actions stored in memory (Decety and Ingvar, 1990; Jeannerod, 1999). Our findings specify this formula by showing that during action observation, the premotor, parietal, and temporal components of this network differ with regard to their sensitivity of object-implied actions: Unlike IPL and pMTG, PMv was only sensitive for the exploitation of competing implied actions, but not for the mere number of implied actions. While IPL and pMTG reflected the action options both as evoked by the sight of the objects (bottom-up) and as competition resolved by frontal biasing signals (top-down), PMv was indifferent with regard to the former: it showed for the number of action codes effect for the interaction between, but not for the conjunction of, object-compatible and object-incompatible actions.

In order to understand and interpret this finding, it helps to consider three converging results in the present study: PMv was present in both object-compatible and object-incompatible action (conjunction contrast, Figure 5), more pronounced in object-incompatible as in object-compatible actions (masked direct contrast, Figure 5) (H3), and driven by object-evoked action options in object-compatible but not in object-incompatible actions (interaction of action codes and object compatibility, Figure 4B). This data pattern suggests that PMv not only registers object-evoked action representations, as aIPL and pMTG do, but also dynamically applies these internal action representations, either in order to adapt to, or to predict, the ongoing action (cf. Schubotz, 2007).

As to the parietal activation revealed in the interaction contrast, it was found to extend from anterior to posterior IPS in the right hemisphere. Why was pIPS activity so pronounced for the right hemisphere? Mruczek et al. (2013) recently reported that tools evoke stronger responses than non-tools in an anterior intraparietal region. Authors suggest that posterior IPS encode features common to any graspable object (including tools), whereas anterior IPS integrate this grip-relevant information with “experience-dependent knowledge of action associations, affordances, and goals, which are uniquely linked to tools” (Mruczek et al., 2013, p. 2892). Coordinates of activation maxima in posterior IPS were most closely located to those related to macaque area MIP (Grefkes and Fink, 2005). MIP is suggested to be involved in coordination of hand movements and visual targets (Eskandar and Assad, 2002), particularly in transforming the spatial coordinates of a target into a representation that is exploited by the motor system for computing the appropriate movement vector (Cohen and Andersen, 2002). Interestingly, these computations take place even in advance of the motion execution itself (Johnson et al., 1996) and hence point to a role of MIP in the detection of movement errors and their correction already on the basis of internal models (Kalaska et al., 2003). More recent studies specify this region as providing tactile information to circuits linking anterior intraparietal to ventral premotor regions, giving on-line feedback needed for goal-directed hand movements (Gardner et al., 2007a,b).

This computational profile was perfectly reflected in the increase of activity in this region reported here, when pragmatic object-implied constraints on expectable manipulations could be integrated with the currently unfolding action. Also the notion of detection of movement errors and their correction on the basis of internal models (Kalaska et al., 2003) fits very well to the present finding, as our parametric contrast pinpointed the competition load between action options. Thus, when multiple action options were implied by the perceived object or object set, and hence represented as multiple internal models of potentially observable manipulations, pIPS may contribute to the detection of discrepancies between expected and observed manipulations.

To finally address activation in the mid-insula, this region relays tactile information from the somatosensory cortex to the frontal cortex (Burton and Sinclair, 2000). Activity was located at the posterior short insula gyrus, which is delimited by the precentral and central insular sulci. This dysgranular region has connections to SI and SII (cf. Guenot et al., 2004 for review). Together with SII and SMG, the mid-insula is suggested to play a crucial role in tactile object recognition, and to integrate somatosensory information to provide a coherent image of an object appropriate for cognitive action (Reed et al., 2004; Milner et al., 2007). Since we found this region to positively co-vary in activity with the number of object-implied expectable manipulations, but only when the observed action matched one of them, we speculate that the enhancement of the matching action comprised also a tactile representation of the observed object manipulation.

### Observing object-incompatible actions: objects evoke action options that do not foster action recognition

In the present study, videos showing object-incompatible actions (i.e., pantomimes with incompatible objects) were employed as a control condition that served to tell apart effects that could be only due to the sight of objects (common to object-compatible and incompatible actions) from effects that could be only due their manipulation (different between object-compatible and incompatible actions). Objects and object sets were always reminiscent of valid action options, both in object-compatible and object-incompatible actions. Moreover, object-incompatible and object-compatible actions were presented randomly intermixed and each occurred with equal probability of 0.5. Together, these design features provoked, as intended, an initial analysis of object information and an attempt to match the observed actions on one of the automatically evoked action code.

To be sure, object-incompatible action is certainly more than just some kind of “incomplete” action, and there are positive effects of object-incompatible action, i.e., activations that come in addition to what we see during observation of object-compatible action. We have investigated these effects elsewhere (Schubotz and von Cramon, 2009). Replicating these findings, object-compatible and object-incompatible action observation yielded highly similar activation patterns in the resting contrast, with significant differences only in the emphasis of different parts of the Action Network (cf. conjunction in Figure 5). Thus, differences in our parametric analyses in object-compatible and object-incompatible action could not be due to principally absent activations in the object-incompatible condition, i.e., “positive effects” in the object-compatible action condition cannot be just due to “negative effects” in object-incompatible actions.

It is important to note that humans are perfectly able to decode actions from object-incompatible manipulations, even from early childhood on (Fein, 1981). Also in the present study, participants performed as well in object-incompatible trials (4.8% errors, 1232 ± 69 ms reaction time) as in object-compatible action trials (5.8% errors, 1192 ± 61 ms reaction time). Saying that participants failed to exploit object-evoked action representations in the case of object-incompatible actions thus does not mean that they failed in the task, but rather, that their strategy had to be adapted according to the stimulus. Object-incompatibility was revealed by a mismatch between the currently active action codes and the actually observed manipulation. As object information was invalid here, observers had to entirely rely on the analysis of hand movements when trying to decode the currently pursued action. In accordance with this suggestion, and replicating a previous study (Schubotz and von Cramon, 2009), we found that the entire Action Network engaged in manipulation recognition (PMv, aIPS, pMTG) enhanced for object-incompatible as compared to object-compatible actions (cf. Hypothesis H3).

However, we also reasoned that action codes should not result in any significant effect on object-incompatible actions since they cannot help to constrain the recognition process. However, the novel and striking finding here was that activity in an area comprising left pSTS and TPJ did increase with the NAC. More specifically, this activation was located in the horizontal posterior segment of the pSTS, extending toward the ascending posterior segment, and hence comprised a temporal-parietal-occipital junction (BA 37, 39 and 19) (Figure 4D and blue spot in Figure 4B). Activation was left-lateralized, corresponding to the processing of information from the right visual field, and, in the present study, the dominant (right) hand of the actor. This fits well with the experimental setting as, to an observer, motion and posture of the dominant hand is more informative than that of the non-dominant hand; the latter typically holds and stabilizes the object while the former performs the relevant manipulations. Focus on the right visual field was also indicated by increased activation in left cuneus (cf. Machner et al., 2009).

These effects of an increasing NAC were only found for object-incompatible actions and indicated that, although object information was in fact not usable here, the expectations of particular hand movements announced by the objects still affected further stimulus analysis. Note that the more action options were evoked by the object, the lower were the constraints on the to-be-expected manipulations. At the same time, the probability increased that the actually observed manipulation eventually matches one of the pre-activated actions: When you expect one out of three potential actions to occur, a fourth and unexpected action is more difficult to detect than in a case when you expect exactly one specific action to occur.

With this in mind, we take left pSTS activation to reflect the intensified focus on the hand's movements in an attempt to decode the displayed action. Interestingly, activation extended posteriorly and dorsally into TPJ. Due to its particular functional profile in attentional orienting as well as in mentalizing paradigms (Decety and Lamm, 2007; Mitchell, 2008), TPJ has been discussed to have a “where-to” functionality in analogy with the spatial “where” functionality of the dorsal stream (Van Overwalle, 2009). That is, it responds to externally generated behaviors with the aim of identifying the possible end-state of these behaviors (cf. discussion in Van Overwalle and Baetens, 2009). Note that the “end-state” of behavior can be read as being actually related to the physical body, as TPJ is related to the sensation of the position and the movement of one's own body (Blanke et al., 2004). Our findings corroborate this interpretation as they showed TPJ activation to proportionally increase with the number of expectable end-states of the unfolding action. Importantly, TPJ activation was not specific to or indicative of object-incompatible (pantomime) perception in general, as the TPJ effect was only found for the action code parameter in object-incompatible actions, whereas it was absent in the direct contrast object-incompatible vs. object-compatible action.

### Concluding remarks

The faculty of understanding what other persons are doing is based, among other factors, on the analysis of object and manipulation information. The present study shows that the action-observing brain accurately extrapolates the expectable actions from the objects that the actor is handling, and, when detecting a match between these expectable actions and the actually observed one, subsequently reinforces the matching action against the competition of the remaining but unobserved actions. These findings impressively reflect that object-evoked actions constrain the recognition process in action observation

### Conflict of interest statement

The authors declare that the research was conducted in the absence of any commercial or financial relationships that could be construed as a potential conflict of interest.
